# Henoch–Schönlein Purpura (IgA Vasculitis) in Association with Thyrotoxicosis

**DOI:** 10.1155/2021/6669653

**Published:** 2021-05-22

**Authors:** Mojgan Sanjari, Mohammadreza Shakibi, Moeinadin Safavi

**Affiliations:** ^1^Department of Endocrinology, Afzalipour Medical Faculty and Endocrinology and Metabolism Research Center, Institute of Basic and Clinical Physiology Sciences, Kerman University of Medical Sciences, Kerman, Iran; ^2^Rheumatology Department, Afzalipour Medical Faculty and Physiology Research Center, Institute of Neuropharmacology, Kerman University of Medical Sciences, Kerman, Iran; ^3^Pathology Department, School of Medicine, Kerman University of Medical Sciences, Kerman, Iran; ^4^Pathology Department, School of Medicine, Tehran University of Medical Sciences, Tehran, Iran

## Abstract

Graves' disease is the most common cause of hyperthyroidism, which is characterized by thyroid antibodies and the following clinical manifestations: goiter, ophthalmopathy, and pretibial myxedema. On the other hand, Henoch–Schönlein purpura is an IgA-mediated small-vessel vasculitis. Review of the literature showed a relationship between propylthiouracil overdose and the following Henoch–Schönlein purpura (IgA vasculitis) as a side effect. The patient was a 31-year-old woman with a chief complaint of tremor and significant weight loss who contracted pruritic palpable purpura during her disease course. Then, she underwent the treatment of hyperthyroidism by methimazole which intensified her cutaneous lesions. The diagnosis of Henoch–Schönlein purpura (IgA vasculitis) was confirmed after skin biopsy. Finally, she was treated with colchicine, prednisolone, and radioiodine ablation, which caused her lesions to disappear. The temporal priority of pruritic palpable skin lesions to hyperthyroidism treatment with methimazole suggested that Henoch–Schönlein purpura (IgA vasculitis) was related to hyperthyroidism and was intensified by antithyroid agents in this patient.

## 1. Introduction

Graves' disease is the most common cause of hyperthyroidism, which is characterized by thyroid antibodies (TSI, antimicrosomal, antithyroglobulin, and ANA) and the following clinical manifestations: goiter, ophthalmopathy, and pretibial myxedema. [1] On the other hand, Henoch–Schönlein purpura is an IgA-mediated small-vessel vasculitis, which is characterized by the following criteria (2 of 4 is 87% sensitive and 89% specific) [2]: (1) palpable purpura, (2) age ≤20 at disease onset, (3) bowel angina, and (4) biopsy showing granulocytes in the walls of arterioles and venules.

Review of the literature showed a relationship between propylthiouracil overdose and the following Henoch–Schönlein purpura (IgA vasculitis) as a side effect. [3] It has been well documented that propylthiouracil could induce antineutrophil cytoplasmic antibody (ANCA)-positive vasculitis, which predominantly affects small vessels. [4, 5] However, the causal relationship between Henoch–Schönlein purpura (IgA vasculitis) and Grave's disease or antithyroid drugs has been remained unclear yet [6].

## 2. Case Report

**Figure 1:**
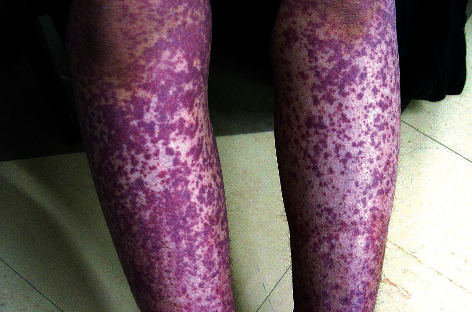
Palpable purpura with more intensity on the lower extremities.

The patient was a 31-year-old woman with a chief complaint of hand tremor and significant weight loss (20 kg during 5 months). She also developed pruritis 5 months later, which led to palpable purpuric rash of both the legs and arms extended to the palms and soles during 2 weeks (Figure 1).

**Figure 2:**
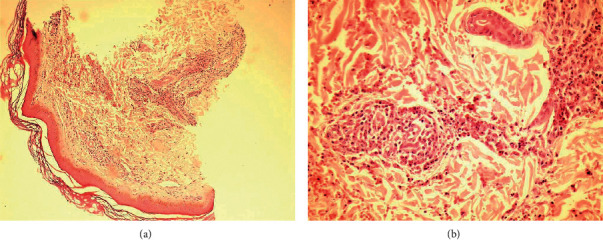
Perivascular neutrophilic infiltration, impinging vessel walls, RBC extravasation, and nuclear dust with endothelial cell plumping in the dermis (hematoxylin and eosin, 40x and 100x magnification, respectively).

More evaluation of the patient by lab tests revealed mild anemia (Hb = 11.3 g/dL) and hyperthyroidism (T4 > 24.86 *µ*g/dL, T3 = 483 ng/dL, and TSH = 0.01 mIU/mL). Thus, the patient underwent the treatment with methimazole thereafter. During the disease course, the purpuric lesions extended to the abdominal skin. Therefore, she was referred to a dermatologist for a more comprehensive workup. According to his opinion, a skin biopsy was performed with the impression of vasculitis. The pathology report showed hyperkeratosis of the *epidermis* and perivascular neutrophilic infiltration, impinging vessel walls, RBC extravasation, and nuclear dust with endothelial cell plumping in the dermis. All these findings were strongly suggestive of Henoch–Schönlein purpura (IgA vasculitis) (Figures 2(a) and 2(b)).

Based on the findings, a complete autoimmune antibody study was performed which yielded the following results: anti-ds-DNA = 40.7 (<100 negative), ANA = 1/20(up to1/10), ANA (pattern) = homogenous, IgA = 3.3 g/l (0.86–3.2), c-ANCA = 0.7 RU/ml (<20 negative), p-ANCA = 1.9 RU/ml (<20 negative), and cryoglobulin = negative.

The result of urine analysis was negative for hematuria and proteinuria. Further lab data including lipid profile and renal and liver function tests were all in the normal range. After consultation with a rheumatologist, treatment with colchicine (10 mg) and prednisolone (100 mg) was started which caused the skin lesions to disappear. Gradual tapering of prednisolone to 25 mg could control the cutaneous lesions. Nevertheless, a lower dose of prednisolone could not resolve the lesion. Therefore, the patient received 10 mCi radioiodine due to the possibility of an allergic reaction to methimazole. Cutaneous lesions disappeared after radioiodine therapy. Three months later, laboratory tests revealed hypothyroidism (TSH = 98 *µ*g/dL) after radioiodine therapy. Therefore, levothyroxine was prescribed for her. Seven months later, skin lesions recurred and laboratory findings showed concomitant hyperthyroidism (TSH = 0.005 *µ*g/dL). Thus, levothyroxine dose was tapered to half and led to skin lesion resolution. Thereafter, the patient was euthyroid, and cutaneous lesions did not recur.

## 3. Discussion

Traditionally, vasculitis is a well-known side effect of Graves' disease treatment with antithyroid drugs such as propylthiouracil and methimazole. It manifests as a systemic sickness with several cutaneous presentations from erythema to necrosis. Leukocytoclastic vasculitis along with fibrinoid necrosis of vessel walls is the most common histopathologic findings in this phenomenon [7]. The pathogenesis of antithyroid drug-associated vasculitis is unclear yet. However, induction of antineutrophilic cytoplasmic antibodies (ANCA) such as antimyeloperoxidase by antithyroid drugs has been proposed as a possible underlying cause. [8].

During the recent decade, few cases of Henoch–Schönlein purpura (IgA vasculitis) have been reported in patients with Graves' disease with or without usual doses of antithyroid drugs suggesting Graves' disease as a triggering factor for Henoch–Schönlein purpura (IgA vasculitis). [9] On the other hand, few cases of Henoch–Schönlein purpura (IgA vasculitis) have been reported with subsequent development of autoimmune thyroiditis or transient hyperthyroidism and a possible immunologic relationship between these two conditions has been postulated [6, 10].

In the described case, the temporal precedence of Henoch–Schönlein purpura (IgA vasculitis) to treatment beginning with methimazole in the background of Graves' disease suggested that these two conditions are associated. However, treatment with methimazole augmented the vasculitis lesions in this case. It is very interesting that vasculitis relapsed under thyrotoxicosis caused by L-thyroxin overdose. Therefore, there are two episodes of hyperthyroidism of different causes, both leading to a vasculitis flare. These findings may show that hyperthyroidism per se irrespective of pathogenesis may cause vasculitis in susceptible individuals. Indeed, the vasculitis resolved after radioiodine therapy and after dose reduction of L-thyroxin, which strongly supports that view.

In conclusion, Henoch–Schönlein purpura (IgA vasculitis) might be occasionally related to thyrotoxicosis and can be intensified by antithyroid agents.

## References

[1] Cooper D. S. (2003). Hyperthyroidism. *The Lancet*.

[2] Mills J. A., Michel B. A., Bloch D. A. (1990). The American college of rheumatology 1990 criteria for the classification of Henoch‐Schönlein purpura. *Arthritis & Rheumatism.*.

[3] Lee J. E., Jeon C. H., Chang D. K., Lee M.-S., Oh H. Y. (2006). Henoch-Schönlein purpura associated with propylthiouracil overdose. *Nephrology Dialysis Transplantation*.

[4] Dolman K. M., Von Dem Borne A. E. G. K., Goldschmeding R. (1993). Vasculitis and antineutrophil cytoplasmic autoantibodies associated with propylthiouracil therapy. *The Lancet*.

[5] Harper L., Cockwell P., Savage C. O. (1998). Case of propylthiouracil-induced ANCA associated small vessel vasculitis. *Nephrology Dialysis Transplantation*.

[6] Shin J. I., Park J. M., Lee J. S., Kim D. H., Jeong H. J. (2005). Development of graves’ disease during cyclosporin treatment for severe Henoch-Schönlein nephritis. *Nephrology Dialysis Transplantation*.

[7] Pietszkowski N. C., Carvalho G. A. D., Souza H. N. D. (2007). Auto-imunidade ANCA (anticorpo anti-citoplasma de neutrófilos) positiva induzida por propiltiouracil: relato de caso e revisão da literatura. *Arquivos Brasileiros de Endocrinologia & Metabologia*.

[8] Ahmed K., Rao S., Simha V. (2010). Antineutrophil cytoplasmic antibody-positive vasculitis in a patient with graves disease: cross-reaction between propylthiouracil and methimazole. *Endocrine Practice*.

[9] Shin J. I., Park J. M., Kim J. H. (2006). Association of Henoch-Schönlein purpura and graves’ disease. *Nephrology Dialysis Transplantation*.

[10] Garcia-Porrua C., Gonzalez-Gay M., Botana M., Sanchez-Andrade A. (2000). Henoch-Schönlein purpura in adults and autoimmune thyroiditis. *The Journal of Rheumatology*.

